# Efficient Illumination for Microsecond Tracking Microscopy

**DOI:** 10.1371/journal.pone.0107335

**Published:** 2014-09-24

**Authors:** David Dulin, Stephane Barland, Xavier Hachair, Francesco Pedaci

**Affiliations:** 1 Department of Bionanoscience, Kavli Institute of Nanoscience, Delft University of Technology, Delft, The Netherlands; 2 Université de Nice Sophia Antipolis, Institut Non-Lineaire de Nice, CNRS UMR 7335, Valbonne, France; 3 BBright, Rennes, France; 4 Centre de Biochimie Structurale, CNRS UMR5048, UM1, INSERM UMR1054, Department of Single-Molecule Biophysics, Montpellier, France; University of Manchester, United Kingdom

## Abstract

The possibility to observe microsecond dynamics at the sub-micron scale, opened by recent technological advances in fast camera sensors, will affect many biophysical studies based on particle tracking in optical microscopy. A main limiting factor for further development of fast video microscopy remains the illumination of the sample, which must deliver sufficient light to the camera to allow microsecond exposure times. Here we systematically compare the main illumination systems employed in holographic tracking microscopy, and we show that a superluminescent diode and a modulated laser diode perform the best in terms of image quality and acquisition speed, respectively. In particular, we show that the simple and inexpensive laser illumination enables less than 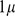
s camera exposure time at high magnification on a large field of view without coherence image artifacts, together with a good hologram quality that allows nm-tracking of microscopic beads to be performed. This comparison of sources can guide in choosing the most efficient illumination system with respect to the specific application.

## Introduction

In optical microscopy, it is today of key interest to combine the high spatial resolving power of microscopes with the ability to detect temporally fast dynamics. In particular, an increasing number of applications based on video microscopy require to detect and track, with nanometer and microsecond resolution, the motion of scattering, non-fluorescent, micro- and nano-particles. Examples include single-molecule biophysics techniques, such as magnetic tweezers [1], [2], (holographic) optical tweezers [3]–[5] and tethered particle motion [6]–[9], together with micro-rheology [10], [11], holography [12]–[14], and micro-fluidic devices [15], [16]. Several implementations of these techniques employ the spatial distribution of the light scattered from microscopic particles to track their motion with nm-resolution or to reconstruct their shape [17]–[22]. These measurements can be used to obtain information about the particle local environment [10], the mechanical behavior of single molecules tethered to the particles [20], and the action of enzymes [23].

In general, when fast dynamical processes (in particular out of equilibrium) have to be detected by a camera, the exposure time of the sensor must be minimized to avoid blurring, while the acquisition rate should be maximized to sample correctly the dynamics. The development of fast CCD and CMOS cameras allows today microsecond exposure time and, consequently, tens to hundred kHz sampling rate (i.e. 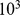 to 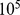 frames per second) to be reached. At these extreme acquisition speeds an important parameter is the quantity of light which arrives from the sample at the sensor. Therefore a major limiting factor for further development of fast microscopy is the efficiency of the illumination system.

The obvious candidate for high flux illumination is of course the laser. However, speckle or other artifacts resulting from spatial and temporal coherence of laser sources often limit their practical use. For this reason, less coherent sources are usually used. The systems employed for microscope illumination include the widespread incoherent lamps and light emitting diodes (LED) [23], [24]. Less commonly employed, superluminescent diodes (SLD) [2], supercontinuum sources [25], and random lasers [26] have also been used. Interestingly, one illumination system with reduced effective spatial and temporal coherence based on a modulated diode laser has been proposed [27] and used at high magnification [28]. Each source may be more or less suitable to a particular application depending on the specific requirement of the measurement to be performed and the best balance between emitted power, spectral properties, possibility of coupling the emitted light with external optical components, setup complexity, generated heat, size and cost has to be found.

Holographic tracking microscopy implies particular illumination requirements in addition to simply high power. In fact, temporal coherence is detrimental to image quality due to possible multiple reflections in the imaging path, but spatial coherence is needed for 3D object tracking. In the following, we compare systematically the different illumination sources and show that the approaches based on SLD and diode laser give the best performances in terms of image quality and acquisition speed, respectively. In particular, illumination by an off-the-shelf single transverse mode semiconductor laser with MHz modulation of bias current enables image acquisition at microsecond exposure time, providing the image quality required for particle tracking with nm-resolution. The technique (which reduces the temporal coherence but preserves the spatial coherence of the laser source) cancels out most of the coherence artifacts while it preserves the particle hologram required for 3D tracking. We assess the efficiency of the laser illumination scheme in comparison to LED, white lamp and SLD illumination and demonstrate its performance by tracking a microsphere at 1 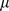
s exposure time. This acquisition speed (obtained with the cheapest of the illumination sources we tested) in our setup is 70 times faster than the one obtained with a SLD, and three order of magnitude faster than the one obtained with a white lamp and LED illumination.

## Results

### Laser illumination and coherence effects

The use of laser light for efficient illumination suffers from the inherent drawback given by its coherence and well defined polarization state: a severe noise in the light spatial distribution arises as soon as different parts of the laser beam interfere with each other. On the other hand, if these coherent effects can be sufficiently reduced, a diode laser becomes an optimal source for fast microscopy illumination because of its high power density, low losses and dissipated heat, small size, low cost, and because it gives the possibility of light collimation and coupling with other optical components, and of choice of wavelength to reduce photo-toxicity at the sample and maximize sensor efficiency.

Methods to decrease the coherence of lasers have been extensively investigated [29], [30], mainly with the goal of producing images free of speckles, e.g. intensity noise produced by laser interference from random surfaces. Several different solutions have been proposed which rely on the average reduction of both spatial and temporal coherence in diode lasers [27], [28], [31]–[33]. We recall that the degree of temporal coherence is measured by the interference which occurs between two temporally delayed copies of the beam, as in a Michelson interferometer, while spatial coherence is measured by the level of interference between two laterally shifted (but not delayed) regions of the beam, as measured in a double slit (or Young) interferometer.

**Figure 1:**
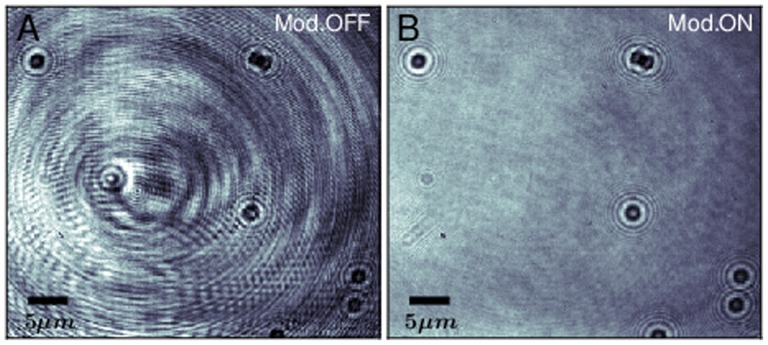
Laser illumination: effect of the current modulation. A) Beads of 1 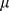
m diameter stuck on the glass surface are illuminated by the free running laser biased above threshold (at a current of 96 mA). The coherent noise severely degrades the image quality. B) Same field of view as in A), with bias current modulated by a sinusoidal signal of 80 mA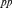, 2 MHz. Exposure time  =  20 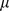
s.

In the tracking microscopy applications we consider here, it is important to recognize that a degree of spatial coherence is required to maximize the contrast of the scattering pattern of the particle and, as a consequence, to improve the accuracy in determining particle position and shape. Polarization effects, on the contrary, are not expected to play a crucial role [34]. While spatial coherence can in principle give rise to speckle formation, in our experiments the main source of noise is not originated from it. The noise is visible in fig. 1A as large interference fringes when we image 1 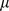
m beads stuck on a glass surface immersed in water and illuminated by the laser diode. Injecting a MHz modulation in the driving current of the diode laser, we produce a fast shift of the laser frequency, effectively reducing the temporal coherence of the laser at the time scale of the camera shutter, leaving the spatial coherence unaffected. The modulation efficiently eliminates the interference noise, while it leaves unchanged the scattering patterns of the particles, as it is visible in fig. 1B. We conclude that the coherent artifacts present in fig. 1A originate from multiple reflection interferences (likely from the internal surfaces of the microscope objective), whose positions are more sensitive than diffraction upon changes in wavelength and can be averaged out in one exposure time.

We note that laser current modulation has long been an obvious approach for laser illumination applications, but in the MHz range it cannot have any meaningful impact on speckle formation [27] since it provides only moderate spectral broadening. When speckles are not an issue, i.e. in absence of random diffusive surfaces, the MHz modulation has remarkable and unique advantages. In fact, due to moderate effective spectral broadening, it preserves diffraction features which are useful for detection and hologram tracking while cancelling out almost completely the multiple reflection artifacts which are typical of coherent illumination and detrimental to image quality. Due to these distinctive features, it emerges as an excellent technique in the specific context of fast tracking microscopy.

### Comparison of illumination systems

In this work we consider the following sources: 1) the free running diode laser, 2) the diode laser driven by a modulated current source, 3) the amplified spontaneous emission (ASE) of the same diode, obtained at bias currents below the laser threshold, 4) one LED, 5) one SLD, and 6) a fiber-coupled white lamp.

**Figure 2:**
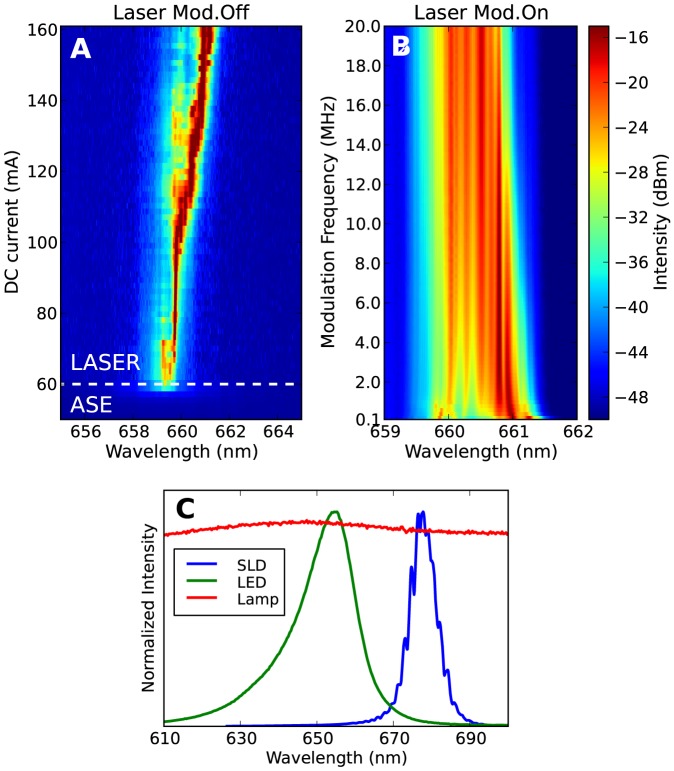
Optical spectra of the illumination sources considered and effect of laser current modulation. A) Optical spectrum of the free running laser diode as a function of the DC bias current. The figure is divided between laser emission (above the threshold current of 60 mA) and amplified spontaneous emission (ASE) below threshold. B) Spectrum of the modulated laser as a function of the modulation frequency (laser DC current: 120 mA, AC modulation: 120 mA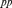, square wave). Each optical spectrum is integrated over a 0.5 s time-window. The color code is the same in A and B. C) Normalized spectra of the SLD, LED, and white lamp (whose spectrum is flat in the visualized region).

In Fig. 2A we show the optical spectrum of the free running diode laser, as a function of the bias DC current. Below the threshold current 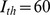 mA, the ASE spectrum is also visible. Overall the laser spectrum displays a red-shift of about 2 nm upon increase of the bias current. This effect is exploited to enlarge the spectrum by current modulation. The spectrum of the modulated laser is shown in Fig. 2B, as a function of the modulation frequency. Due to the typical thermal rise time in semiconductor lasers under current modulation (0.01 to 1 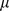
s [35], [36]), modulation frequencies in the MHz regime induce large temperature variations in the semiconductor medium. The consequent thermal shift in emission wavelength can be integrated in one camera exposure time of 1 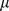
s (the shortest allowed by our CMOS camera), creating a virtual multimode source with decreased temporal coherence. The typical broadening of the diode emission spectrum is about 1 nm with a square wave modulations of 2–20 MHz, which brings the laser current from 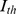 to 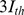. This broadening does not change significantly in the range of frequencies explored, as it is visible in Fig. 2B, or with the shape of the modulation. A frequency within the range of 2 to 8 MHz is optimal both for maximum spectral broadening and averaging over the minimum camera exposure time. The emitted average laser power is 120 mW and the beam can be easily collimated and focused. When driven by a steady current below threshold, the ASE is evidently weaker than the laser (Fig. 2A), nonetheless it is a source we consider because of its low temporal coherence (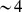 nm bandwidth) and easiness of beam coupling and manipulation with external optical components.

A LED is a semiconductor medium where gain occurs without optical feedback, so coherent laser emission is impeded and the temporal coherence is low (bandwidth of 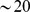 nm, Fig. 2C). The main drawback is the difficulty in manipulating or focusing the highly divergent beam, which has the consequence of high losses and therefore demands for high power (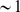 W) if high acquisition rates are sought. A SLD is a semiconductor cavity with only one highly reflective surface, a design in between a LED and a laser. The one we use displays 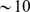 nm bandwidth (Fig. 2C), and power of 5 mW distributed in an asymmetric beam. The white source we test has a very broad spectrum, in the range 360–2400 nm (shown in part in Fig. 2C), and consists of a 5 W halogen lamp coupled to a mm-size optical fiber bundle.

**Figure 3:**
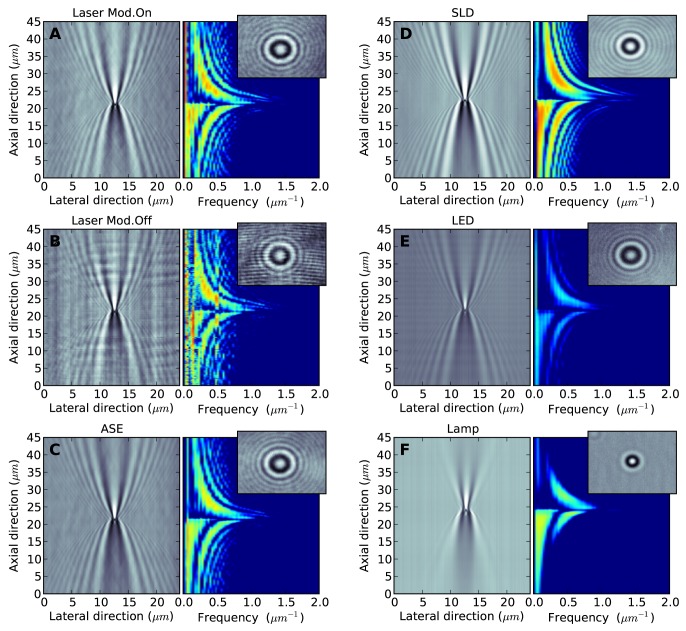
Scattering patterns from 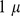
m bead. In each panel (A–F) we show, for each source, the projection of the 3D hologram on the axial and lateral direction in the left panel, and its Fourier representation in the right panel. The inset shows the real image obtained at axial position 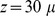
m. The grey and color scale is the same for all the panels. Exposure times: A) and B) 2 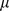
s; C) 3 ms; D) 70 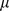
s; E) 15 ms; F) 6 ms.

To better compare the different illumination sources for tracking applications, we characterize in Fig. 3 the holograms obtained illuminating the same microscopic object with the different sources. For each illumination, we image a single bead (1 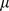
m diameter) stuck to the glass surface of the flow cell and immersed in water, changing the axial position (i.e. along a direction parallel to the laser propagation) of the focal plane. We reconstruct in this way the 3D hologram of the bead, and show its section in the axial and lateral directions in the left panels of Fig. 3A–F. For each illumination (delivering its maximum power), the images have been acquired using the minimum exposure time which gives a good image in terms of visibility of the diffraction rings. In this way we can compare directly the efficiency of the illumination via the minimum exposure time that could be reached. Using the lasing diode (modulated and not modulated, in Fig. 3A and 3B respectively), an exposure time of 2 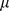
s could be attained. The diode ASE required a longer exposure of 3 ms (Fig. 3C). Using the SLD we could reach 70 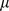
s (Fig. 3D), while for the LED (Fig. 3E) and the white lamp (Fig. 3F) it was necessary to keep the shutter open for 15 ms and 6 ms respectively. This shows the high illumination efficiency of the modulated laser and the SLD illuminations, which allow acquisitions in the microsecond range. In the right panels of Fig. 3A–3F we plot the Fourier representation of the corresponding bead hologram section discussed above. The presence of high harmonics for a given axial position is an additional indicator for fringe visibility. Considering the laser, it is evident that the modulation is required to eliminate the coherent noise, which compromise the quality of the images. The images where diffraction rings are most visible and spatial noise is minimum, are obtained with the SLD. The LED gives also low noise but less contrast. The extremely low coherence of the white source greatly decrease the pattern visibility with respect to all other more monochromatic sources we consider, and as a consequence the presence of the microscopic object becomes barely detectable when it is observed more than 10 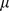
m from the focal plane, while its scattering pattern remain visible for many tens of 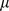
m using the other sources.

**Figure 4:**
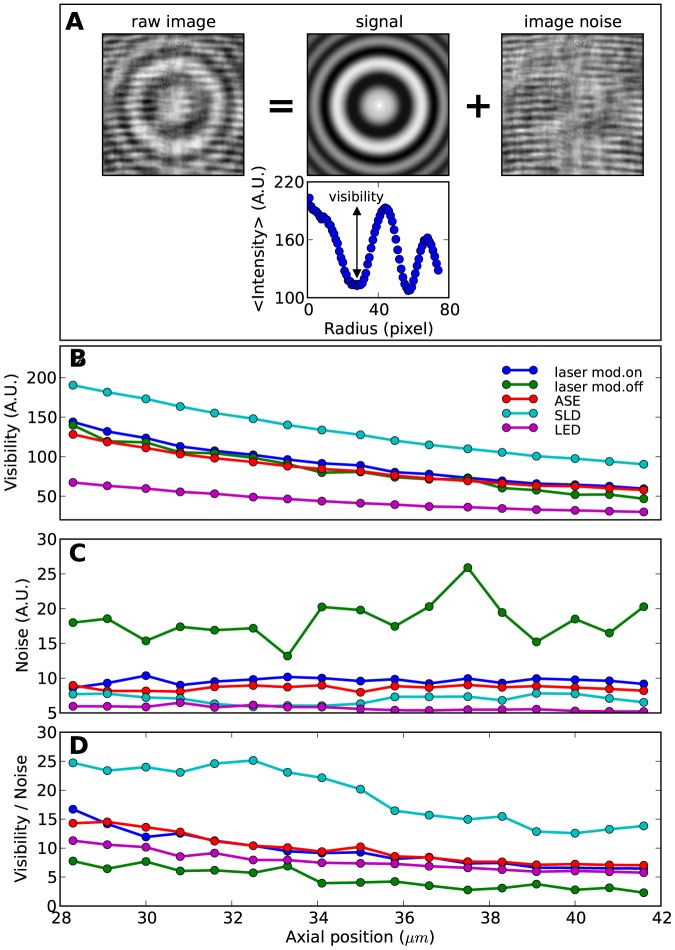
Quantifying fringe visibility and image noise. A) Example showing the results of the angular average algorithm used to extract signal, fringe visibility and local image noise from the raw images. The raw image shown is obtained with the unmodulated laser at 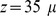
m. The signal is obtained averaging the raw image rotated in 100 steps around its center (determined with sub-pixel resolution). The image noise is obtained subtracting the signal from the raw image. B) Fringe visibility (defined in the radial intensity profile by the difference of the second maximum with the first minimum), C) noise (defined as the standard deviation of the image noise), and D) visibility-to-noise ratio are shown for the different sources at different axial z-positions.

To compare quantitatively the image quality for the different sources (excluding the white source) at different axial positions, we need to separate from the raw images the signal produced by the scattering object and, at the same position, the local noise due to the illuminating source and camera detection. We extract the fringe visibility and local image noise from the raw images using the following algorithm (depicted in Fig. 4A). Using the circular symmetry of the imaged bead, we compute an angular average of the raw image, effectively averaging out the image noise. This is done rotating and averaging the image in 100 steps around its center (determined with sub-pixel resolution [20]). The resulting signal can be subtracted from the original raw image to obtain the image noise. The visibility, shown in Fig. 4B as a function of axial position, is measured as the contrast of the first maximum in the radial intensity profile of the signal image. The noise, shown in Fig. 4C, is the standard deviation of the retrieved noise image. In Fig. 4D, the visibility-to-noise ratio clearly shows that the best images are obtained using the SLD, followed by the modulated laser, the ASE and the LED. Not surprisingly, the unmodulated laser is characterized by the lowest signal-to-noise ratio. Importantly, the signal to noise ratio is found to be two to three times larger for the modulated laser than for the unmodulated laser.

**Figure 5:**
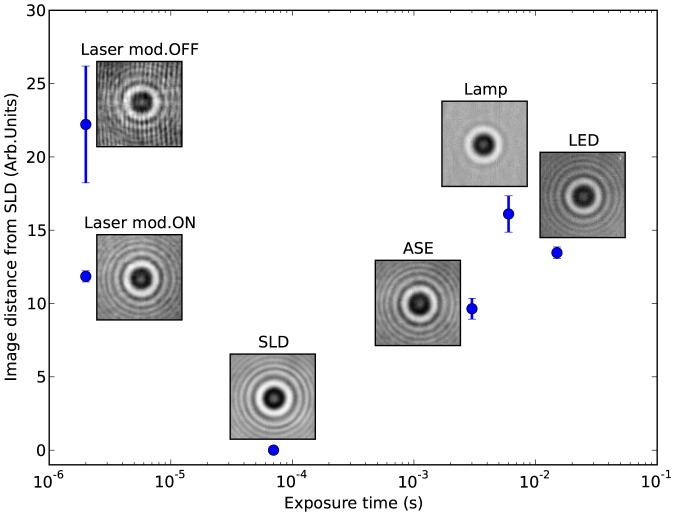
Comparison of the illumination sources. The six sources are compared in terms of attainable exposure time and relative image quality. The latter is quantified relatively to the SLD source, which gives the best image quality. In the vertical axis we plot the average distance of the images obtained with each source from the images obtained with the SLD (see Methods).

Having found that the SLD produces the best images in terms of fringe visibility, in Fig. 5 we use the images obtained with this source as reference, and we compare the different sources in terms of their distance to this reference together with the attainable exposure time. At each 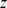 position, we quantify the distance between the SLD reference image and the images obtained with the other sources (see Methods). In the vertical axis of Fig. 5, for each source, we plot the distance from the SLD image averaged along 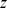. This classification shows that the image quality obtained with the modulated laser compares well with the other sources, with the advantage of shorter exposure time.

**Figure 6:**
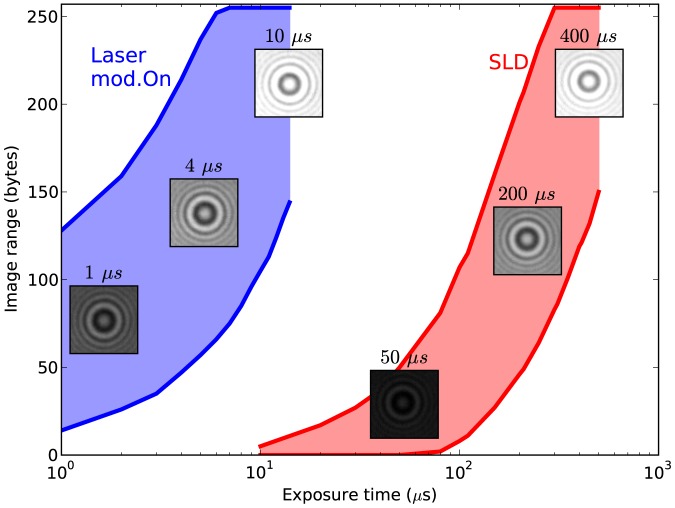
Comparison of the modulated laser and SLD illumination. Image range (maximum and minimum pixel values) are shown as a function of the exposure time for the modulated laser (blue) and the SLD (red) illuminating the same object (one 1 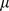
m bead out of focus). The insets show example images obtained at the exposure time indicated; their grey levels are all fixed within the interval (0, 255) to show under-exposure and saturation. The two sources were focused to illuminate evenly the same field of view, and delivered maximum intensity (laser: 120 mW, sinusoidal modulation of 3 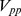 at 2 MHz; SLD: 5 mW).

In Fig. 6 we further quantify the difference between the images obtained with the modulated laser and the SLD. Both sources were delivering the maximum power (120 mW and 5 mW respectively) and were focused to illuminate evenly the same field of view (69×86 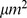). As a function of the exposure time, we plot the range of the images of the same microscopic bead obtained with the two sources. Exposure time shorter than 50 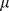
s cannot be used with the SLD, while with the modulated laser 1 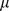
s is attainable and faster exposures are in principle possible. We estimate that to reach 1 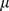
s exposure time the laser delivers on the camera plane an order of 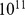 photons/(s pixel). The image obtained in 1 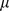
s with 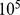 photons/pixel spans 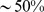 of the sensor dynamic range. We find that the same image range is reached with the SLD with an exposure time of 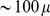
s. Likely, in addiction to its 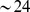 times lower emission, the SLD suffers from more coupling losses due to higher beam divergence. It is worth noting that with a more focused illumination both sources can reach shorter exposure, at the expenses of an uneven illumination across the field of view (in an optimized illumination geometry the SLD was shown to allow exposure times shorter than 28 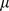
s [2]).

### Tracking with 1 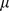
s exposure time with laser illumination

**Figure 7:**
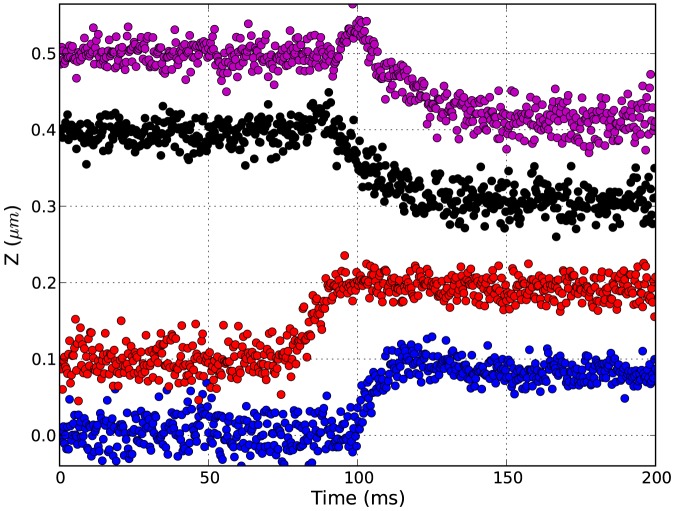
Tracking at 1 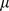
s exposure. Z trajectories of a stuck bead tracked while moving the objective in steps of 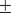
100 nm. The traces are vertically offset for clarity. Acquisition rate: 3000 fps, exposure time: 1 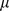
s.

To demonstrate the actual feasibility of fast tracking using the modulated laser source, we show in Fig. 7 the result of bead tracking setting the exposure time at 1 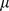
s. The stuck bead is illuminated by the modulated laser and the focal plane is rapidly shifted using a piezo stage that controls the position of the objective in steps of 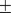
100 nm. The 3D tracking algorithm first finds the x and y position of the center of the diffraction pattern, then compares the ring pattern of the acquired image with a previously built library of calibration images to find the best z position [20]. In Fig. 7 we show four raw trajectories of the z position of the bead where the imposed step is well sampled and resolved. We set the acquisition rate to 3 kHz, while in principle rates up to few tens of kHz are possible using our CMOS camera at this exposure time, depending on the size of the region of interest used on the sensor area.

## Discussion

We have compared the performance of different illumination sources used in tracking video microscopy. The comparison takes into account the specificities of fast tracking applications: the need of a degree of spatial coherence in order to observe clear diffraction patterns, the need to obtain images free of spatial noise due to the coherence of the source, and the need to minimize the camera exposure time, which require high photon flux from the sample to the sensor.

The two sources which allowed in our setup exposure times in the microsecond regime are the laser and the SLD. The minimum exposure time of the camera (1 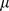
s) could be reached only by the modulated laser, which can allow even shorter exposure. With this exposure, we have shown that the laser illumination produces images that can be used to track the common 1 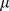
m beads employed in many applications. We note that the minimum exposure time attainable is the relevant parameter to compare because, together with the electronics of the camera, it limits the maximum acquisition rate (in frames per seconds).

Although the image quality obtained with the modulated laser is of slightly inferior quality with respect to the SLD source (a factor of 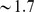 is found between the signal-to-noise ratios of the images obtained with the two sources), the diffraction rings are essentially preserved (while they are washed out with incoherent illumination) and the multiple reflection artifacts are almost completely absent. Therefore, for tracking applications (which we demonstrate to be robust with respect to minor image degradation) laser diode modulation appears to be the best approach for microparticle tracking with microsecond exposure time, while SLD illumination provides the best signal-to-noise images at longer acquisition (50–100 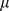
s). The precise physical effect of current modulation on laser emission properties is out of scope of the present paper. Optimization of modulation parameters could lead to better image purity at even faster acquisition.

## Materials and Methods

The sources used are the following. Laser: HL6545MG (Thorlabs), power of 120 mW, nominal wavelength of 660 nm. Superluminescent diode: SLD-260 MP2 T09 (Superlum), 5 mW maximum power, 670 nm nominal wavelength. LED: M660L3 (Thorlabs), 700 mW typical power, 660 nm nominal wavelength. Lamp: HL-2000 Tungsten Halogen Light, 5 W bulb power, 360–2400 nm wavelength range. The diode laser and the SLD are both mounted on a laser diode mount with integrated temperature control (LDM9T, Thorlabs). A voltage generator (Agilent 33210A) directly modulates the laser current via a 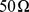 bias tee.

The setup consists of a home-built inverted microscope based on a 100x 1.40 NA oil immersion objective (Nikon), mounted on a piezo stage (P-726.1CD Physik Instrumente). On the camera plane, one pixel (14 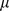
m) corresponds to 67 nm in the sample plane. The flow cell is simply made with two sandwiched cover slips separated by double side tape, and filled with water. Super-paramagnetic beads (Myone, 1 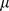
m diameter) adhere aspecifically on the bottom glass kept at high temperature and remain stuck on the surface after cooling. The fast CMOS camera (CL600x2 Optronis) is connected by Full Camera Link to a FPGA frame grabber (NI PCIe-1473R, National Instruments) and 8 or 10 bit images can be continuously streamed to the solid state disks of the computer in RAID0 configuration without losses or interruptions. All hardware control and real time tracking of beads is implemented in software written in Labview (National Instruments). The laser, LED and SLD are all mounted 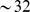 cm above the flow cell and focused on the sample with a single lens (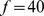 mm) carefully adjusted to illuminate the full field of view. The laser was not collimated before focusing. In a magnetic tweezers setup, we have checked that the illumination geometry of the laser and SLD is compatible with the presence of magnets with a gap of 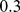 mm at a distance of 0 to 4 mm from the flow cell top surface. Therefore forces in the range of 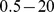 pN should be possible on Myone beads tethered to DNA constructs, without severely affecting the illumination efficiency. Using the white lamp source, we put the fiber few mm from the top of the flow cell, without using a lens.

The image distance plotted in Fig. 5 is defined as follows. If 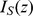 is the bead image obtained at position 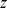 using the source 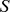  =  (unmodulated laser, modulated laser, ASE, SLD, LED, and white lamp), its distance from 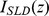 is 
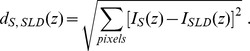



For each source 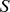, in the vertical axis of Fig. 5 we plot the average and standard deviation of 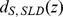 along 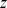, excluding the images of a region of few microns around the 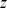 position where the bead is at focus, where the rings disappear and the differences between images become larger.
